# I-gel Laryngeal Mask Airway Combined with Tracheal Intubation Attenuate Systemic Stress Response in Patients Undergoing Posterior Fossa Surgery

**DOI:** 10.1155/2015/965925

**Published:** 2015-07-26

**Authors:** Chaoliang Tang, Xiaoqing Chai, Fang Kang, Xiang Huang, Tao Hou, Fei Tang, Juan Li

**Affiliations:** Department of Anesthesiology, Anhui Provincial Hospital, Anhui Medical University, No.1 Swan Lake Road, Hefei 230036, China

## Abstract

*Background*. The adverse events induced by intubation and extubation may cause intracranial hemorrhage and increase of intracranial pressure, especially in posterior fossa surgery patients. In this study, we proposed that I-gel combined with tracheal intubation could reduce the stress response of posterior fossa surgery patients. *Methods*. Sixty-six posterior fossa surgery patients were randomly allocated to receive either tracheal tube intubation (Group TT) or I-gel facilitated endotracheal tube intubation (Group TI). Hemodynamic and respiratory variables, stress and inflammatory response, oxidative stress, anesthesia recovery parameters, and adverse events during emergence were compared. *Results*. Mean arterial pressure and heart rate were lower in Group TI during intubation and extubation (*P* < 0.05 versus Group TT). Respiratory variables including peak airway pressure and end-tidal carbon dioxide tension were similar intraoperative, while plasma *β*-endorphin, cortisol, interleukin-6, tumor necrosis factor-alpha, malondialdehyde concentrations, and blood glucose were significantly lower in Group TI during emergence relative to Group TT. Postoperative bucking and serious hypertensions were seen in Group TT but not in Group TI. *Conclusion*. Utilization of I-gel combined with endotracheal tube in posterior fossa surgery patients is safe which can yield more stable hemodynamic profile during intubation and emergence and lower inflammatory and oxidative response, leading to uneventful recovery.

## 1. Introduction

Systemic and cerebral hemodynamic changes caused by extubation and emergence from anesthesia may endanger neurosurgical patients and increase the risk of postoperative intracranial hemorrhage and cerebral edema and may even result in the requirement of reoperation [1].

Orotracheal intubation has been proven to be a reliable method for securing the airway and is considered to be the standard technique for intraoperative management of the airway during neurosurgery. During the procedure, the patient's head is covered and hidden under the surgical field and held in a position that is not convenient to control the airway. Therefore, intubation that can ensure adequate ventilation for long period may be the best choice. However, endotracheal intubation induces more intense hemodynamic effects and physical stress than those caused by the use of a laryngeal mask airway (LMA). During emergence from anesthesia and extubation, these differences are even more intense that can lead to increases in cerebral blood flow, intracranial pressure, and regional brain oxygen saturation (rSO_2_) [2].

Generally, stress is defined as the hormonal and metabolic changes that follow any injury to the biological system. Such stress response is characterized by the systemic reaction to injury which encompasses a wide range of endocrinological, immunologic, and hematological effects [3]. The severity of stress response during surgery affects not only patient outcomes but also health care system. The plasma concentrations of *β*-endorphin (*β*-EP), cortisol (Cor), and blood glucose level (BG) are a reflection of stress sensitive indicators during anesthesia and surgery, and significant fluctuations in serum glucose levels accompany the stress response of surgery. In the recent decade, the brain has been regarded as an organ, which is susceptible to inflammation or immune activation, and also thought to be largely affected by systemic inflammatory and immune responses and oxidative stress [4, 5]. Pathological inflammatory states can have far ranging clinical effects and negatively influence a patient's neurological outcome [6–8]. Cytokines regulate the acute phase response. Several cytokines are released during periods of stress, including interleukin-6 (IL-6), interleukin-8 (IL-8), and tumor necrosis factor-alpha (TNF-*α*) [9]. Studies have shown that surgical and anesthesia manipulation-induced sympathetic activation and oxidative stress may be the main factors that lead to hypertension, which could trigger the sympathetic nervous system and seems to be injurious to the patients, especially for neurosurgical patients [10]. Modification of the circulating hormone Cor, *β*-EP, BG level, inflammatory cytokine, and oxidative stress may be necessary to improve the surgical outcome [11].

In recent years, LMA is widely used in clinical practice due to its simple operation, low stimulation, and light stress reaction [12–14]. I-gel without sac is made of special medical grade thermoplastic elastomer and does not need to be inflated. It has a unique baffle that prevents the epiglottis folding and airway obstruction to reduce the likelihood of airway obstruction [15, 16]. I-gel without sac can be inserted into the stomach tube to prevent regurgitation and aspiration, and its massive air duct may help with the endotracheal tube placement. Due to the special position of posterior fossa surgery, simply placing a LMA is not conducive to ensure adequate ventilation that may lead to the accumulation of carbon dioxide and increases of intracranial pressure. I-gel combined with bronchial occlude for thoracic surgery has been reported [17]. However, the clinical use of I-gel combined with endotracheal tube for posterior fossa surgery has not been explored.

We compared the safety characteristics, systemic hemodynamic variables, the stress and inflammatory response, oxidative stress, and cough incidence during the induction and emergence of posterior fossa surgery patients receiving general anesthesia either used this new I-gel combined with endotracheal tube technique or used a traditional endotracheal tube airway technique in a prospective randomized clinical trial. Primary outcome measures were ease of perioperative stress and inflammatory response, and oxidative stress, airway management, and incidence of coughing.

## 2. Material and Methods

This prospective, randomized, clinical trial was approved by the Ethics Committee of Anhui Provincial Hospital, Anhui Medical University (file number 2011/07), and registered at Chinese Clinical Trial Registry (ChiCTR) with registration number ChiCTR-OOC-14005623.

### 2.1. Patients

Informed consent was obtained from all the patients. Sixty-six patients of either sex with the American Society Anesthesiologists physical status I-II, aged between 18 and 60 years, undergoing posterior fossa surgery under general anesthesia from the neurosurgery department of our center were recruited. Types of surgery included 12 cases of cerebellar hemisphere tumors, 6 cases of cerebellar vermis tumor, 12 cases of cerebellopontine angle tumors, 6 cases of fourth ventricle tumor, 25 cases of acoustic neuroma, and 5 cases of slope tumor. All patients were informed of the experimental protocol and purpose of the study.

Exclusion criteria included heart diseases, endocrine system diseases, and uncontrolled high blood pressure detected during preoperative assessment; predicted difficult airway, risk of bronchial aspiration (e.g., gastroesophageal reflux disease or lower cranial nerve palsy) and patients who need respirator for assisted ventilation after operation; obesity (BMI > 30 kg/m^2^); I-gel inserted unsuccessfully more than twice, patients with contraindications for early emergence based on anesthetic or surgical criteria or as a result of complications developing during surgery was also withdrawn from our study.

### 2.2. Study Design and Anesthesia Procedure

Four senior anesthetists and three high qualification residencies performed all the operations. The patients were randomized to the two study groups by random number table method, which was prepared by an unwitting statistician, tracheal tube intubation (Group TT) and I-gel facilitated endotracheal tube intubation (Group TI) (*n* = 33).

Patients took omeprazole (20 mg) on the ward the night before surgery. In the operating room, patients were premeditated with penehyclidine hydrochloride (0.5 to 1 mg,* i.m.*). Standard monitoring consisted of five-lead electrocardiography (ECG), oxygen saturation (SpO_2_), mean arterial pressure (MAP), arterial and central venous pressures, and depth of anesthesia as assessed by BIS. Then all patients received hydroxyethyl starch 130/0.4 (Voluven) 8~10 mL/kg and were supplemented with oxygen (4 liter/min) via an O_2_ nasal cannula. Dexmedetomidine was given at 0.6 *μ*g/kg and then changed into 0.4 *μ*g/kg/h for maintenance after 15 min. Before the start of anesthesia induction, an arterial line was inserted under local anesthesia and located the transducer probe at the level of the foramen of Monro; the line remained in place till the patient shifted to the surgical ward. 100% oxygen was pre-oxygenated before induction, which was delivered through a facial mask for no less than 3 minutes. General anesthesia was provided in the supine position with intravenous propofol (Cp 3.0–4.0 *μ*g/mL) and remifentanil (Cp 3.0–4.0 ng/mL) delivered through a target controlled infusion system (ALARIS MK III, CareFusion, Switzerland) and rocuronium bromide (0.6–0.8 mg/kg). Manual facemask ventilation was continued for no less than 4 minutes until the jaw was relaxed and the BIS was less than 50. The endotracheal tube was inserted with the help of direct laryngoscope in Group TT (using ID 7.5 or 8 mm, for women or men, resp.). For patients in Group TI, the I-gel was inserted according to the manufacturer's instruction (using size 3 or 4, for 30~60 kg or 50–90 kg, resp.). Then the endotracheal tube was inserted through the I-gel's air duc under the guidance of the 2.8-mm Flexible Intubation Videoscope (TIC-SD-II; UESCOPE, China) (using ID 6.0 or 6.5 mm, for size 3 I-gel or 4 I-gel, resp.). The successful placement criteria of I-gel were confirmed by sides of the thoracic move ups and downs properly and lung respiratory sound was auscultated symmetrically, no sound was detected when airway pressure > 20 cmH_2_O, ETCO_2_ waveform was normal, and the insertion of stomach tube was easy.

A ventilator (S/5; Datex Ohmeda, Helsinki, Finland) was connected immediately afterwards. Respiratory parameters were set at: VT 10 mL/kg, RR 12 times/min, and FiO_2_ 60% to maintain ETCO_2_ in the normal range. 1% sevoflurane was inhaled and the target-controlled anesthesia system (TCI) was used to administer propofol and remifentanil to maintain the BIS between 40 and 60 and the MAP and HR variation not exceed 20% of the baseline values.

Patients were injected with tramadol 1.0 mg/kg and azasetron (10 mg) through the intravenous line after fixed skull flap. In Group TI, after suturing the scalp, we pulled out the endotracheal tube, stopped the TCI of anesthetics to make it possible for the patient to quickly emerge from anesthesia. We then shifted the patient to supine position and used the I-gel to continue to maintain the patient's ventilation until the patient was awake and then pulled out the I-gel. In Group TT, after suturing the scalp, the patient still under general anesthesia was shifted to supine position without modification of any anesthetic drug administration. Then we continued the ventilation with the same parameters as described earlier. TCI of anesthetics were then stopped to allow the patient quickly emerge from anesthesia. Before the patient resumed spontaneous breathing and responded to simple commands, gentle manual ventilatory assistance was provided. The endotracheal tube was then removed. The criteria of pulling out the endotracheal tube or I-gel was: (1) the recovery of consciousness, muscle tension returned to normal, fist clenched strongly according to the instruction; (2) steady spontaneous breathing, ETCO_2_ < 45 mmHg, tidal volume > 7 mL/kg; (3) the SPO_2_ > 97% after stopping to receive oxygen for 5 min; (4) the frequency of spontaneous breathing < 24 times/min; (5) cough and swallowing reflex recovered. The modified observer's assessment of alertness/sedation/(MOAA/S) score reached 3 as assessed according to the criteria: (0 (asleep) = no response to painful trapezius squeeze; 1 = responding only after painful trapezius squeeze; 2 = responding only after mild prodding or shaking; 3 = responding only after name is called loudly, repeatedly, or both; 4 = Lethargic response to name spoken in normal tone; 5 (alert) = responding readily to name spoken in normal tone) [18].

### 2.3. Outcome Measures

Hemodynamic variables (MAP and HR), were recorded at eight time points: baseline, before dexmedetomidine infusion (T0); before anesthetic induction (T1); endotracheal tube intubation (T2); 3 min after intubation (T3); end of surgery but before awakening (Group TT) or before endotracheal tube removed (Group TI) (T4); after patients entered the PACU (T5); and throughout emergence from anesthesia at 1, 5, 15, and 30 minutes after extubation or I-gel removal (according to group assignment) (T6-9). Respiratory variables (including peak airway pressure and end-tidal carbon dioxide tension) were recorded during mechanical ventilation, and arterial blood gases were also recorded. Blood pressure elevation (Blood pressure > 140/90 mmHg or increases more than 20% value), tachycardia (HR > 100 beats/min or an increase of >30 beats/min from baseline), choking cough occurrence during the recovering period of anesthesia were recorded and timely treated. Nicardipine, or esmolol was administered at a dose where the anesthesiologist considered appropriate to maintain the patient with stable vital signs and drug doses were recorded. Choking cough reflex degree is divided into: (1) No choking cough, breathe evenly; (2) Mild choking cough, separate a choking cough; (3) Moderate choking cough, choking cough < 30 s; (4) Severe choking cough, choking cough lasts 30 s or higher. Operation time, spontaneous breathing recovery time (the time that the patients resumed breathing after stopping giving anesthetics), eye opening time (the time that the patients opened their eyes after stopping giving anesthetics), extubation or I-gel removal time (the time that extubation in Group TT or I-gel removal in Group TI), and the MOAA/S score when extubation or I-gel removal were recorded.

### 2.4. Blood and Sputum Processing and Analyses

Four milliliter of venous blood was collected at following time points: T0, T1, T3, T5, and T6. One drop of blood was taken to measure blood glucose level (BG). The rest was added to tubes without anticoagulant, perfectly still until the serum separation, the serum precipitated was taken with centrifugal to centrifuge at 4000 rpm in 4°C for 10 min, and then the supernatant was sucked out to place in −80°C cryogenic refrigerator to wait to test *β*-EP and Cor at T0, T1, T3, T5, and T6; IL-6, IL-8, TNF-*α*, MDA and Superoxide Dismutase (SOD) at T0 (pre-operation) and T6 (post-operation). *β*-EP and Cor were assayed by enzyme-linked immunosorbent assay (ELISA) (2-CAT; Elabscience Biotechnology Co., Ltd). IL-6, IL-8, TNF-*α*, MDA and SOD levels in plasma were measured in the Immune Surveillance Laboratory at The Research Institute at Anhui Provincial Hospital using the Immulite automated chemiluminometer (Siemens Healthcare Diagnostics, Deerfeld, IL). All assays were performed according to manufacturer's instructions.

### 2.5. Statistical Analysis

The demographic characteristics were compared using a two-sample *t* test for continuous data. The *χ* ^2^ test was used to analyze categorical variables. Data are presented as Mean ± SD, Mean ± SEM or as count (%). Student *I* test and ANOVA were used for unpaired quantitative variables. All reported *P* values were two-sided, and *P* values less than 0.05 were considered significant. The statistical analyses were performed with SPSS Statistics 13.0 software.

## 3. Results

**Figure 1:**
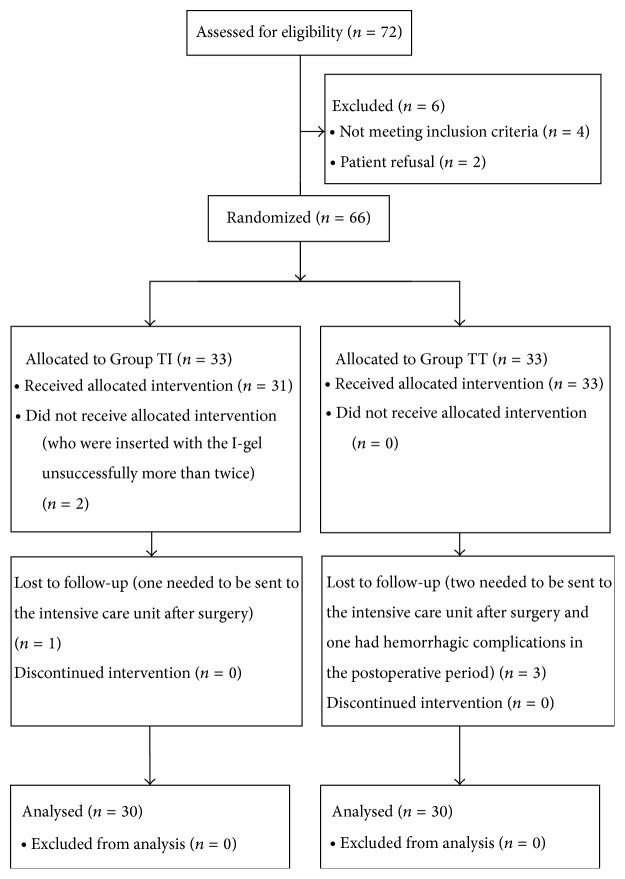
Flow diagram of patient recruitment.

Flow diagram of patient recruitment was shown in Figure 1.

Patient and procedural characteristics of both groups are shown, respectively, in Table 1. There were no significant differences between groups at baseline.

**Figure 2:**
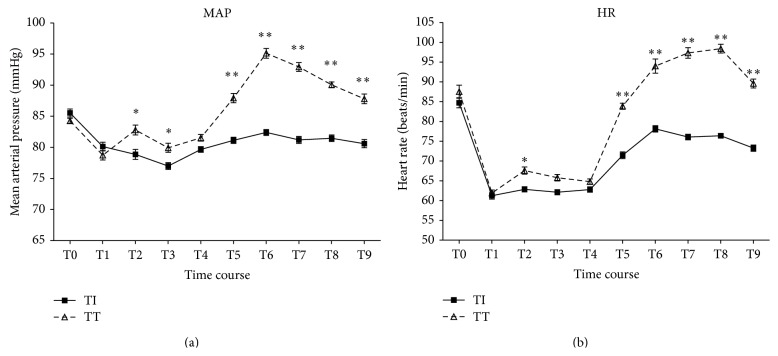
Changes in mean hemodynamic variables in patients with a tracheal tube intubation (Group TT) or an I-gel facilitated endotracheal tube intubation (Group TI) during the study; bars indicate the SEM. Both groups had significant reduction in MAP and HR from their respective baseline values till the end of the surgery. Patients in Group TI had a greater fall in comparison to Group TT over the time period of the observation (*P* < 0.0001). During emergence from anesthesia, MAP and HR in Group TT were significantly higher than that at baseline (*P* < 0.0001). And the intergroup differences were also significant (^*∗*^ *P* < 0.05; ^*∗∗*^ *P* < 0.0001). Baseline (T0); before anesthetic induction (T1); endotracheal tube intubation (T2); 3 min after intubation (T3); end of surgery but before awakening (Group TT) or before endotracheal tube removed (Group TI) (T4); after patients entered the PACU (T5); and throughout emergence from anesthesia at 1, 5, 15, and 30 minutes after extubation or I-gel removal (according to group assignment) (T6-9).

No differences were found at baseline measurements of HR and MAP between the two groups. Both the groups had significant reduction in MAP and HR from their respective baseline values till the end of the surgery, however, patients in Group TI had a greater fall in comparison to Group TT over the time period of the observation. Of note, MAP and HR on emergence from anesthesia in Group TT were significantly higher than that at baseline (highest of the significant *P* < 0.0001). Intergroup comparison of MAP and HR at similar time intervals during intraoperative period, Group TI had a lower MAP and HR, especially at the time of endotracheal tube intubation (*P* < 0.05). Similarly, Group TI had lower MAP and HR on emergence from anesthesia in PACU than those in Group TT. The highest mean between-group difference was 12.73 mmHg and 22.0 beats/min (*P* < 0.0001). Most of differences were narrowed 30 minutes after extubation or I-gel removal (Figure 2).

**Figure 3:**
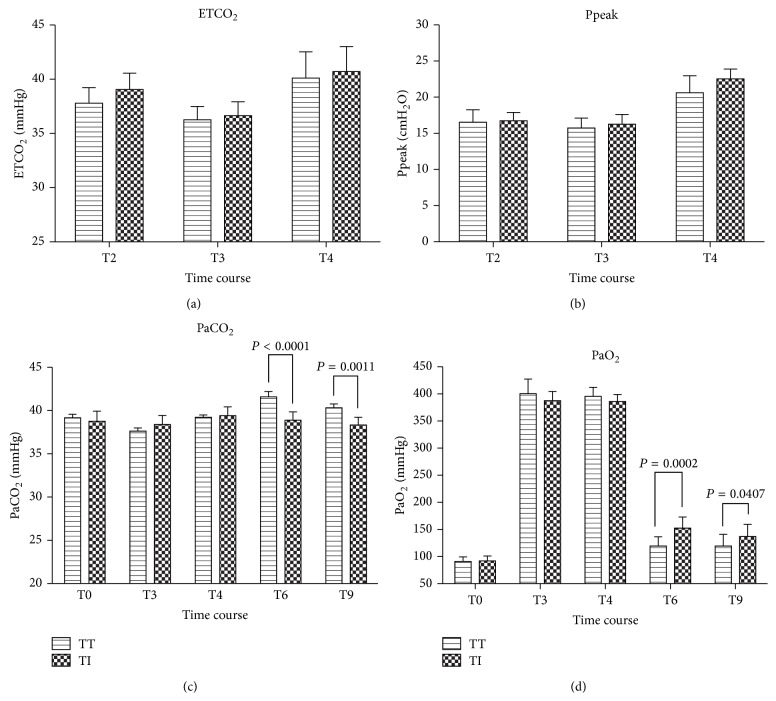
Changes in ETCO_2_, Ppeak, PaCO_2_, and PaO_2_ in patients with a tracheal tube intubation (Group TT) or an I-gel facilitated endotracheal tube intubation (Group TI) over a period of time. Results are expressed as Mean ± SD. No significant difference in ETCO_2_ and Ppeak over time. During emergence, PaCO_2_ and PaO_2_ were better in Group TI than those in Group TI, and the differences were significant. Baseline (T0); endotracheal tube intubation (T2); 3 min after intubation (T3); end of surgery but before awakening (Group TT) or before endotracheal tube removed (Group TI) (T4); and throughout emergence from anesthesia at 1 and 30 minutes after extubation or I-gel removal (according to group assignment) (T6, 9).

Ppeak, ETCO_2_, PaO_2_ and PaCO_2_ were comparable and within normal limits in both groups. Although the abovementioned respiratory parameters tended to be more optimal in Group TT during intraoperative period, the differences did not reach statistical significant (*P* > 0.05). At 1 and 30 minutes after emergence, PaCO_2_ were (38.9 ± 0.95 mmHg) and (38.3 ± 0.89 mmHg) in Group TI in comparison to (41.6 ± 2.39 mmHg) and (40.3 ± 1.68 mmHg) in Group TT, and the differences were significant between groups at both time points (*P* < 0.0001 and *P* = 0.0011, resp.) (Figure 3(c)). Similarly, PaO_2_ at 1 min (152.6 ± 20.52 mmHg) and 30 min (137.3 ± 22.24 mmHg) in Group TI were significantly higher than those (119.5 ± 17.19 mmHg and 119.6 ± 21.65 mmHg, resp. at 1 min and 30 min) in Group TT (*P* = 0.0002 and *P* = 0.0407, resp.) (Figure 3(d)).

**Figure 4:**
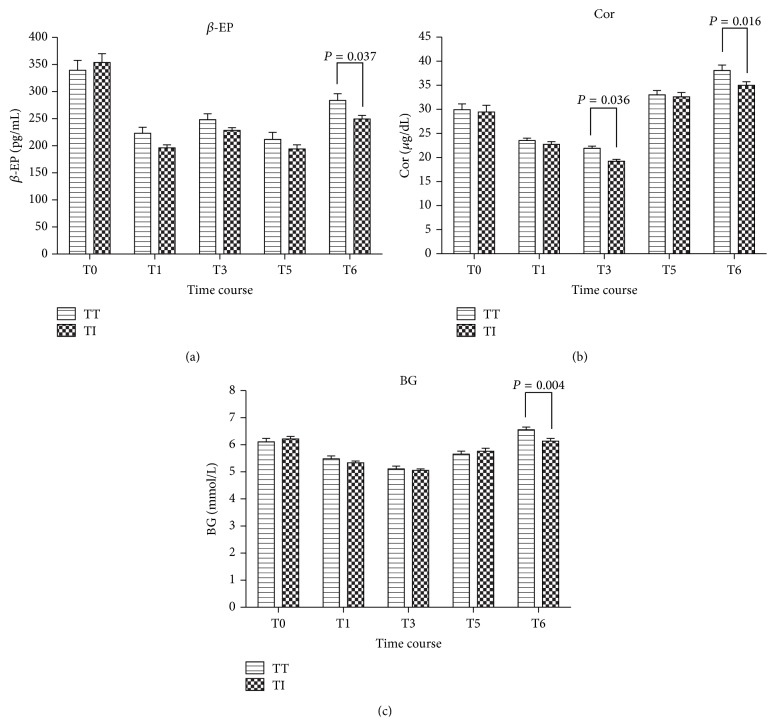
Changes in plasma *β*-EP and Cor concentrations and BG in patients with a tracheal tube intubation (Group TT) or an I-gel facilitated endotracheal tube intubation (Group TI) during the study. Results are expressed as Mean ± SD. Mean concentration was lower during intraoperative period than at baseline in both groups (*P* < 0.0001). They began to rise during emergence, and the Cor concentration even higher than that of baseline (Group TT, *P* < 0.0001; Group TI, *P* = 0.0008). The percent increase tended to be higher in Group TT (*P* < 0.05). Baseline (T0); before anesthetic induction (T1); 3 min after intubation (T3); after patients entered the PACU (T5); and throughout emergence from anesthesia at 1 minute after extubation or I-gel removal (according to group assignment) (T6).

Plasma *β*-EP concentration varied greatly. Mean concentration of *β*-EP was lower during the whole procedure than at baseline in both groups (*P* < 0.0001). Only at 1 minute after extubation, the *β*-EP concentration in Group TI became higher than that in Group TT (*P* = 0.037) (Figure 4(a)). Also, Plasma Cor concentration was lower during intraoperative period than at baseline in both groups (*P* < 0.0001), but it slowly began to rise on emergence from anesthesia and reached its highest value at 1 minute after extubation or I-gel removal, and was higher than its baseline values, respectively, in Group TT and Group TI (*P* < 0.0001 and *P* = 0.0008, resp.). Intergroup comparison, Group TT had higher Cor concentration at 3 min after intubation (*P* = 0.036) and 1 minute after extubation (*P* = 0.016) (Figure 4(b)). BG level was lower during intraoperative period than at baseline in both groups (*P* < 0.0001), it also slowly began to rise to close to baseline on emergence from anesthesia, however, the differences were not significant (*P* > 0.05). The percent increase of BG tended to be higher in Group TT as compared to that in Group TI (*P* = 0.004) (Figure 4(c)).

**Figure 5:**
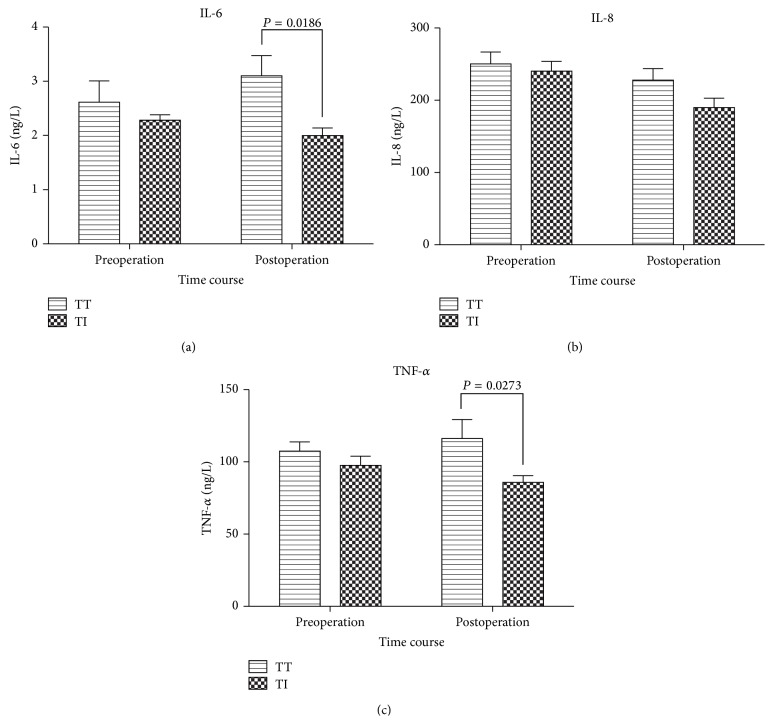
Changes in plasma IL-6, IL-8 and TNF-*α* concentration in Group TT and Group TI. Values are given as Mean ± SEM. Group TT, tracheal tube intubation group; Group TI, I-gel facilitated endotracheal tube intubation group.

Plasma concentrations of IL-6, IL-8, and TNF-*α* during pre-operation and post-operation are displayed in Figure 5. IL-8 level was decreased over time in both groups. IL-6 and TNF-*α* were decreased in Group TI, but increased in Group TT. Intergroup comparison, there were no differences in both experimental groups during pre-operation. However, IL-6 and TNF-*α* were significant lower in Group TI post-operatively. (*P* = 0.0186 and *P* = 0.0273) (Figures 5(a) and 5(c)).

**Figure 6:**
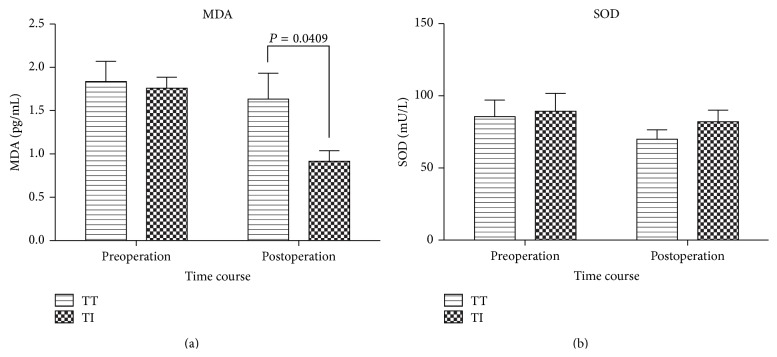
Changes in plasma MDA and SOD concentrations in Group TT and Group TI. Values are given as Mean ± SEM. Group TT, tracheal tube intubation group; Group TI, I-gel facilitated endotracheal tube intubation group.

MDA and SOD levels were decreased over time in both groups, but Group TI had a significant decrease of MDA (*P* = 0.0384, post-operation versus pre-operation). Between-group comparison, MDA levels were significantly higher (*P* = 0.0409) in Group TT during post-operation (Figure 6(a)). SOD activity was greater in Group TI during post-operation. However, no significant difference in SOD levels was observed between the groups (*P* = 0.6263) (Figure 6(b)).

As shown in Table 2, spontaneous breathing recovery time and eye opening time were shorter in Group TI, but the differences were not significant (*P* = 0.084 and *P* = 0.426). I-gel removal time in Group TI (22.0 ± 3.0) in comparison to extubation time in Group TT (19.0 ± 1.0) was significantly difference (*P* < 0.001). However, the MOAA/S score was significantly higher in Group TI at the time of I-gel removal (*P* = 0.032).

Mild choking cough occurred in 12 of 30 patients (40%), moderate choking cough were 6 (20%), and severe choking cough was 1 (3%) in Group TT and only 3 mild choking cough patients (10%) in Group TI (*P* < 0.001). Blood pressure and heart rate increase in Group TT as compared to Group TI. Patients that needed vasoactive drugs for at least once preoperatively are 13 (43.3%) in Group TT versus 2 (6.7%) in Group TI (*P* < 0.001). All Group TT patients known to have controlled chronic hypertension before surgery required pharmacological intervention whereas only one of the 5 known chronically hypertensive patients in Group TI required treatment (Table 3).

No patient in Group TI had hemorrhagic complications during the postoperative period, but one excluded from the study in Group TT due to hemorrhagic complications.

## 4. Discussion

I-gel laryngeal mask airway in combination of tracheal intubation for patients undergoing posterior fossa surgery not only ensured the normal ventilation during intraoperative, but also reduced the hemodynamic impact of intubation and emergence from anesthesia in our study in terms of MAP and HR reduction, the main outcome measure. The incidence of coughing was also lower with I-gel use. As far as we know, this is the first study to compare the effects of I-gel in combination of tracheal intubation for posterior fossa surgery patients, who are particularly susceptible to hemodynamic changes.

Anesthesiologists had to pay more attention to the unattenuated hemodynamic responses, caused by orotracheal intubation, which is an extremely invasive procedure performed at induction of anesthesia. According to Kovac's study about hemodynamic responses to laryngoscopy and endotracheal intubation, laryngoscopy has the maximal increase in BP, and endotracheal intubation has the maximal increase of HR [19]. During conventional laryngoscopy, the maximum force transmitted by a laryngoscope blade onto the base of the tongue is considered to be exceptionally invasive [19], and this stimulation even may be as high as approximately 40 Newtons [20, 21]. This is also verified in our study, the hemodynamic variables as well as MAP and HR were lower in Group TI during intubation. We inserted the endotracheal tube through the I-gel's air duct under the guidance of the Flexible Intubation Videoscope in Group TI. We used the properly seated I-gel as a conduit for endotracheal tube, which can reduce adverse cardiovascular responses due to the avoidance of such a strong stimulus to laryngeal tissues [22, 23]. Moreover, Cros et al. [24] confirmed that tracheal intubation through the ILMA can be used in patients with a difficult airway, and allow for continuous ventilation and oxygenation during tracheal intubation attempts. Also the use of Flexible Intubation Videoscope, reduced the intubation time and improved the accuracy of intubation.

Although the highest mean intergroup difference of 12.73 mmHg in MAP and 22.0 beats/min in HR during emergence from anesthesia may not seem numerically impressive, it would be clinically significant in these posterior fossa surgery patients. We continuously evaluated all our patients for hypertension and tachycardia, and whenever it was identified it was treated with a standard protocol. Nevertheless, most patients in Group TT developed MAP close to 100 mmHg and HR over 100 beats/min, and intergroup differences were statistically significant and as such 43.3% of Group TT patients needed treatments with vasoactive drugs that had probably blunted a more severe rise in blood pressure. On the contrary, only 6.7% patients (2 out of total of 30 patients) in Group TI required vasoactive drug treatment. The 6.5-fold greater need for vasoactive drugs in Group TT reflected a higher incidence of blood pressure surges, which are events that increase the risk of postoperative intracranial hemorrhage. Therefore, we are confident that the differences were also clinically relevant in this setting. In Group TI, after having sutured the scalp, we pulled out the endotracheal tube, stopped the TCI of anesthetics to make the patient could quickly emerge from anesthesia in the operation room. This does attenuate hemodynamic responses, oxygen consumption and stress hormone concentrations as compare with patients in Group TT that were associated with delayed awakening until later in the post anesthesia care unit. Such hemodynamic changes have been reported to be a kind of incentive for intracranial bleeding and cerebral edema. According to a retrospective study of Basali, the incidence of cerebral hemorrhage was 0.77% after craniotomy, and 62% of patients with this complication had developed hypertension in the immediate postoperative period [25]. And other reports have also linked the prior history of hypertension to postoperative hematoma [26]. Our results indicate that in such patients, it can attenuate the rise in hemodynamic responses during emergence from anesthesia by pulling out the endotracheal tube before shifted the patient to supine position and then using the I-gel to continue to maintain the patient's ventilation, as less vasoactive agents were needed in this subgroup. All Group TT patients known to have a controlled history of chronic hypertension before surgery needed pharmacological intervention during emergence but only one of the 5 known controlled-chronically hypertensive patients in Group TI required treatment. Our findings are consistent with those of previous researches of anesthesia convalescence [27], even though we did not design to recruit enough patients to detect differences in the subgroup of patients with controlled chronic hypertension. We think that our results could suggest that the beneficial effects of patients awakening with an I-gel in place would be particularly beneficial in the subgroup of patients with chronic hypertension undergoing craniotomy especially posterior fossa surgery that doctors often fixed the patients' head with the head frame.

Numerous studies have explored methods to reduce the effects of extubation on systemic hemodynamic response. Drugs such as diltiazem/nicardipine [28], esmolol [29], fentanyl [30], dexmedetomidine [31] and lidocaine [32] have been used for this purpose. Remifentanil is the most commonly administration in neurosurgery, which has been suggested as a strategy to smooth emergence generally, and enhance analgesia effect and subsequently reduce hemodynamic impairment during emergence. However, it must be carefully titrated to avoid neurological depression, as well as respiratory depression that may lead to hypercapnia and further hyperemia; otherwise, it may abolish the beneficial effect of hemodynamic control [33]. Smith et al. [34] advocated that postoperative patients with general anesthesia in neurosurgery should be pulled the endotracheal tube under deep anesthesia to avoid the cardiovascular stress reaction. However, the residual effect of sedative, analgesic, and muscle relaxants could lead to insufficient alveolar ventilation, the accumulation of carbon dioxide and hypoxia. Therefore, this method is not suitable for posterior fossa surgery patients. Some scholars [13, 35] also advocated pulling out the endotracheal tube and then making use of the LMA to maintain ventilation at the end of neurosurgery, but the two operations increased the risk of momentary loss of control of the airway. Also, some airway obstruction and the aspiration of gastric content may occur while the airway is unprotected, and we must maintain an adequate depth of anesthesia to ensure the LMA inserting successfully that may delay the patients' awakening. However, patients with anticipated or known difficult airway or at high risk of bronchial aspiration mustn't be recommended to use such replacement techniques [36].

The LMA has been used successfully in some neurosurgical procedures, such as ventriculoperitoneal shunt [37], lumbar spine microsurgery [38], and awake craniotomy [39]. In our study, we also successfully used I-gel combined with endotracheal tube in 30 patients undergoing posterior fossa surgery. It had less effect on hemodynamic response when I-gel was inserted, and also had more stable hemodynamics by the way of inserting the endotracheal tube through the I-gel's air duct under the guidance of the Flexible Intubation Videoscope than that of inserting the tracheal tube using a direct laryngoscope. Although the inner diameter of the trachea was less in Group TI than that of Group TT, there were no significant difference of ETCO_2_, Ppeak, PaO_2_ and PaCO_2_ during intraoperative period between the groups. On the contrary, PaO_2_ and PaCO_2_ were better in Group TI than Group TT at 1 min and 30 min after extubation.

Plasma *β*-EP and Cor concentrations and BG level were lower during intraoperative period than at baseline in both groups. It indicated that general anesthesia could partly limit the perception of stimuli from injury and have little effect on the endocrine and physiological functions. Plasma *β*-EP and Cor concentrations and BG level began to rise, some of them even higher than that of baseline during emergence from anesthesia, and the percent increase tended to be higher in Group TT. It showed that the stress reaction at the time of extubation was greater than that of the intubation, and also the strongest point during the whole process. I-gel combined with endotracheal tube can also reduce the stress reaction level both during intubation and extubtion.

IL-6 is an endogenous pyrogen, which exerts multiple effects that are both beneficial and destructive to CNS cells. IL-8 is a chemokine produced mainly by macrophages and epithelial cells and functions to attract neutrophils towards inflammation sites. TNF-*α* is one of the central mediators of tissue inflammation and proinflammatory during the acute phase of CNS inflammatory. The inflammatory reaction and oxidative stress did not significantly change over time in both groups, which may be explained by the use of dexmedetomidine and propofol. Several studies have reported both dexmedetomidine and propofol could attenuate the inflammation and oxidative stress [40–42]. In our study, there was a significant plasma IL-6, TNF-*α* and MDA decrease during post-operative period in Group TI and a slight increase in reported hemodynamic can be partially explained by the expected increase in hemodynamic after extubtion. Group TT had a higher IL-6, TNF-*α* and MDA levels with a significant increase in hemodynamic during emergence from anesthesia. The decrease of SOD in both groups could be because of the decrease in antioxidant status. And the decrease of MDA in both groups may be due to the use of anesthetics that has antioxidant property.

In our study, the high incidence of cough in Group TT was in contrast with the incidence of 3.6%, which reported in another recent research of patients emerging from anesthesia after craniotomy [43]. This can be explained by our detailed and strict recording criteria of any occurrence of cough during the awakening process. The cough incidence rates in our study were 10% and 63%, respectively, for I-gel and endotracheal tube. We used the I-gel for its' better seal of the airway, good evacuation of gastroesophageal contents, simply guided insertion and especially for its' high tolerance [44]. There were no serious adverse reactions, such as cough, elevated blood pressure, and tachycardia when removal the I-gel in Group TI, and also the patients were very clear for neurosurgeon to do early neurological assessment.

The current study has a number of limitations. It was a randomized, but not double-blinded trial. We arranged an independent observer recorded most of the variables identified, but the observer may clearly identify which device was used. We must also note the small effect of nicardipine on increasing HR, because a certain degree of the observed increase in that variable. In order to reduce its influence on HR, we used esmolol at the appropriate time. In this case, we will not record it as the usage of vasoactive drug. We used the I-gel for about five hours during the surgery, but we simply observed whether the patients had such complications as hoarseness, throat pain, and the damage of the contact parts during the recovery period, we did not have a systematic study and follow-up like Taheri et al. [45]. However, according to our observation, there were no serious complications in Group TI.

In conclusion, I-gel combined with endotracheal tube for ventilation can be effective in preventing the cardiovascular response, attenuating inflammatory and oxidative response, reducing adverse events risk, and improving the quality of anesthesia during intubation and extubation in posterior fossa surgery. Use of the I-gel in this way may be of special interest in patients with a difficult airway or a history of chronic hypertension. It also may provide a novel path for neurosurgical anesthesia airway management.

## Figures and Tables

**Table 1:** Subject and procedure characteristics.

Characteristic	Treatment groups		
	Group TT (*n* = 30)	Group TI (*n* = 30)	*P* value
Age (years)	49 (8)	48 (7)	0.774
Gender (M/F)	18/12	14/16	0.747
Weight (kg)	64 (8)	67 (6)	0.206
Height (cm)	163 (8)	163 (7)	0.867
Procedures			
Cerebellar hemisphere tumors	6 (20%)	4 (13%)	NS
Cerebellar vermis tumor	2 (7%)	2 (7%)	NS
Cerebellopontine angle tumors	6 (20%)	6 (20%)	NS
Fourth ventricle tumor	3 (10%)	3 (10%)	NS
Acoustic neuroma	10 (33%)	13 (43%)	NS
Slope tumor	3 (10%)	2 (7%)	NS
Duration of surgery (min)	335 (20)	338 (23)	0.696

Values are given as Mean ± SD, or number of patients (%).

Group TT: tracheal tube intubation group; Group TI: I-gel facilitated endotracheal tube intubation group.

**Table 2:** Spontaneous breathing recovery time, eye opening time, extubation or I-gel removal time, and MOAA/*S* score.

Characteristic	Treatment groups		
	Group TT (*n* = 30)	Group TI (*n* = 30)	*P* value
Spontaneous breathing recovery time (min)	9.0 (1.3)	8.0 (1.5)	0.084
Eye opening time (min)	11.0 (1.5)	10.0 (2.1)	0.426
Extubation or I-gel removal time (min)	19 (1.0)	22 (3.0)	<0.001
MOAA/*S* score: 5 (alert)/4/3/2/1/0 (asleep)	0/6/20/4/0/0	4/14/12/0/0/0	0.032

Values are given as Mean ± SD.

Group TT: tracheal tube intubation group; Group TI: I-gel facilitated endotracheal tube intubation group.

**Table 3:** Adverse events during the procedure.

Adverse events	Treatment groups		
	Group TT (*n* = 30)	Group TI (*n* = 30)	*P *value
No choke to cough	11 (37%)	27 (90%)	<0.001
Mild choking cough	12 (40%)	3 (10%)	<0.001
Moderate choking cough	6 (20%)	0	1.000
Severe choking cough	1 (3%)	0	1.000
Hypertension	15 (50%)	3 (10%)	<0.001
Tachycardia	5 (17%)	0	1.000
Use of vasoactive drugs	13 (43.3%)	2 (6.7%)	<0.001

Values are given as number of subjects (%).

Group TT: tracheal tube intubation group; Group TI: I-gel facilitated endotracheal tube intubation group.
